# Beyond the cut-off: measurement bias, calibration, and fairness in mild cognitive impairment screening

**DOI:** 10.3389/fmed.2026.1814214

**Published:** 2026-04-10

**Authors:** Waseem Jerjes, Daniel Harding

**Affiliations:** 1 Department of Primary Care and Public Health, Faculty of Medicine, Imperial College London, London, United Kingdom; 2 North End Medical Centre, Hammersmith and Fulham Primary Care Network, London, United Kingdom

**Keywords:** cognitive screening, diagnostic misclassification, diagnostic thresholds, health equity, measurement bias, mild cognitive impairment, screening fairness, threshold governance

## Introduction

Mild cognitive impairment (MCI) is commonly framed as the stage between typical aging and dementia, where decline is detectable yet daily independence is largely preserved. In real-world care, however, this clinical construct is often reduced to a threshold rule: a brief cognitive screening score below a fixed cut-off. The rule is attractive because it is fast, scalable, and seemingly objective. But thresholds are not biological constants; they are decisions made from particular samples, in particular settings. In old age psychiatry, Montreal Cognitive Assessment (MoCA) cut-offs that look “accurate” in studies can lose specificity in practice because of spectrum bias when unusually healthy controls are used to define normal performance (1).

Recent evidence underscores how unstable “the right cut-off” can be. A systematic review and meta-analysis of MoCA for detecting MCI showed that diagnostic accuracy varies widely and that optimal thresholds differ across studies and settings, making the default cut-off a poor substitute for local validation (2). A meta-analysis focused on older adults in China similarly concluded that the optimal MoCA cut-off for screening is context dependent rather than fixed (3). When a score is treated as a gatekeeper for referral, biomarker testing, or therapy access, threshold instability becomes an equity issue rather than a purely psychometric one.

This article is a conceptual Opinion piece rather than a systematic or scoping review. The literature cited was selected purposively to represent the main domains relevant to threshold use in MCI screening: diagnostic accuracy and cut-off instability, demographic and cultural influences on performance, measurement bias and non-invariance, and the downstream consequences of screening decisions for referral and access. The aim is therefore interpretive and framework-building rather than exhaustive evidence synthesis, formal study appraisal, or pooled effect estimation. In the sections that follow, we explain why fixed cut-offs generate predictable misclassification, examine how threshold decisions shape fairness and access, and propose a practical framework for calibrated and auditable threshold use in routine care. In this article, “threshold governance” refers to the explicit design, justification, application, and review of cognitive screening cut-offs, including how they are calibrated, how uncertainty is handled, and how subgroup error and downstream access effects are audited.

Threshold misclassification is patterned because test performance is patterned. Robust demographically adjusted MoCA norms derived from a large multi-site clinical trial illustrate that substantial numbers of cognitively normal older adults can fall below classic cut-offs once age and education are properly accounted for (4). In primary care, where screening decisions are made under time pressure, undiagnosed cognitive impairment remains common and intersects with social and clinical complexity (5). In federally qualified health centers, the prevalence of unrecognized cognitive impairment highlights how delayed detection compounds other inequities in access to preventive and specialist care (6). The argument developed here is that equitable MCI care requires threshold governance and that cut-off use should be made more accountable in everyday clinical pathways.

For conceptual clarity, three levels of concern need to be separated. Measurement bias refers to distortion within the test itself, when scores are shaped by education, language, literacy, culture, sensory barriers, or testing conditions rather than the intended cognitive construct. Diagnostic classification bias refers to the way those scores are converted into decision rules, such that the same cut-off yields uneven false-positive and false-negative rates across groups. Healthcare access inequity refers to the downstream effects of those decisions, when referral, biomarker testing, specialist review, or treatment access become uneven. Threshold governance is intended to address all three levels by improving measurement fit, making classification rules explicit, and auditing pathway consequences.

## Why fixed cut-offs misclassify in predictable, inequitable ways

Cut-offs misclassify because brief cognitive tests measure more than neurodegeneration. They also measure schooling, literacy, language concordance, sensory capacity, and familiarity with timed examiner-led tasks. In older adults with low literacy, comparing common short screens demonstrated that optimal cut-offs differ by instrument and that standard thresholds are not transferable across literacy levels (7). Education-related effects appear in other clinical contexts as well. Among elderly inpatients, years of education influence both Mini-Mental State Examination (MMSE) and MoCA performance, meaning that a single numerical threshold can systematically penalize those with fewer educational opportunities (8). A systematic review focused on low educational and literacy levels concluded that many widely used screening tools are biased by education and that evidence supporting alternatives is uneven, often based on small and context-specific samples (9). Across these studies, the mechanism is educational bias: schooling and literacy shift test performance such that a single numeric cut-off yields systematically higher false-positive “impairment” in those with fewer educational opportunities. In equity terms, fixed cut-offs convert structural disadvantage into apparent disease.

Language and culture amplify these effects. A systematic review of brief cognitive screening in Spanish-speaking adults in Latin America reported that MoCA is widely used but strongly influenced by education and often requires cultural adaptation and education-specific thresholds, while evidence for illiterate and indigenous populations is strikingly limited (10). Globally, MoCA has been translated and culturally adapted into many versions, but the quality and rigor of adaptation varies considerably, raising doubts about cross-cultural fairness without additional validation (11). Even screening tasks assumed to be “simple” are culturally loaded. A systematic review of the Clock Drawing Test found that education, literacy, and other cultural determinants influence performance in most analyses, cautioning against assuming that a low score reflects neuropathology rather than context (12). These data illustrate cultural–linguistic loading as a second mechanism of inequity: when tasks and norms are not aligned with local language and culture, low scores can reflect mismatch rather than pathology.

Psychometrics adds a quieter mechanism: measurement non-invariance. If items change meaning or difficulty across groups or across time, a total score does not represent the same construct for everyone, and fixed cut-offs become unstable anchors. Longitudinal analysis of MoCA items in a Chinese (Cantonese) cohort identified violations of measurement invariance across repeated assessments with different patterns by education group (13). For equity, this matters because longitudinal monitoring is often proposed as a fairer approach; if the instrument is not measuring the same thing across time and context, then apparent “change” can be partly measurement artifact. Clinically, that can mean unnecessary escalation after a small score drop, or misplaced reassurance after a stable score, when the shift is driven by item drift, learning effects, language mediation, or other testing-context factors rather than true cognitive change.

Finally, thresholds misclassify when non-cognitive barriers are mistaken for neurocognitive disease. Clinical cohorts show that cognitive complaints are embedded in multimorbidity. In a memory-evaluation cohort from Lima, potentially reversible metabolic and endocrine abnormalities were common, reminding clinicians that low cognitive scores require medical context and differential diagnosis rather than automatic labeling (14). Socially patterned vascular risk adds another layer. Biomarker-oriented work in elderly Latinx adults at risk of small vessel disease paired MoCA with domain testing and adjusted for education and testing language, illustrating both the feasibility and necessity of context-aware interpretation when cognitive measures are used to infer brain disease (15). This illustrates a comorbidity mechanism: reversible illness, vascular risk, and other clinically actionable factors can depress scores, so cut-off decisions without medical and social context amplify inequity through avoidable misclassification. When thresholds ignore this complexity, they misclassify in the same direction for the same patients.

## Equity harms, race corrections, and the limits of “better” thresholds

Building on the distinction above, this section focuses on diagnostic classification bias and downstream access inequity. Even when a test is widely used, the same cut-off can generate unequal false-positive and false-negative rates across groups, and the consequences of that score can differ because pathways to follow-up, imaging, biomarkers, and specialty care are themselves unequal. Both are visible in debates about interpreting MoCA across racial groups. An analysis explicitly questioned whether clinicians interpret MoCA correctly and demonstrated how a single standard cut-off can differentially classify Black and White adults, reigniting controversy about group-specific thresholds (16). The practical temptation is to “fix” the problem by adjusting the number. The ethical risk is that the adjustment can reify race as biology, obscure the role of educational quality and exposure, and mask structural causes of inequity. To avoid misinterpretation, our position is not that all adjustment is inherently problematic, but that adjustment must be transparent, clinically justified, and secondary to improving the measure itself. Education-related adjustment can be defensible when it captures measurable differences in literacy, educational opportunity, or test familiarity and when its effects on false-positive and false-negative rates are reported openly. Cultural adaptation and validated local-language versions should be preferred over *post hoc* correction whenever feasible, because improving the instrument is ethically stronger than correcting the score after the fact. By contrast, biologically framed race-norming treats race as an intrinsic cognitive modifier and is difficult to justify, particularly when it lowers the probability of further assessment or support for groups already facing structural disadvantage. In practical terms, measured context may be defensible, improved measurement is preferable, and race as a biological proxy is the least defensible basis for threshold adjustment.

Cross-cultural neuropsychology initiatives argue for a more defensible response: measure relevant context rather than correct for race as a proxy. A European consensus statement on cross-cultural neuropsychological assessment emphasizes culturally informed practice, improved measurement of sociocultural variables, careful adaptation and selection of tests, and transparency about uncertainty (17). A related critique proposes performance-based alternatives to race-norming, aiming to preserve diagnostic accuracy without embedding race in the scoring algorithm (18). These positions do not deny group differences; they challenge the idea that the ethically acceptable way to handle them is by hard-coding race.

The evidence base that underpins thresholds is itself inequitable. A review of neuropsychological research reporting found underrepresentation of Black Americans and inconsistent reporting of demographic variables, limiting the validity of norms and cut-offs for populations most affected by disparities (19). When norms are built from unrepresentative samples, “objective” thresholds become structural gatekeepers. Misclassification is also shaped by how MCI is defined. In a racially diverse sample, consensus diagnosis, actuarial neuropsychological criteria, and statistical methods produced different MCI prevalence estimates and different associations with race and longitudinal outcomes, showing that even the “gold standard” used to validate cut-offs is method dependent (20). A systematic review of neuropsychological actuarial criteria concluded that these approaches can show promise in some datasets but may be prone to diagnostic errors in more demographically diverse populations, precisely where equity stakes are highest (21).

Misclassification harms are not abstract. In a latent transition analysis of older adults assessed over three occasions, conventional MCI classification generated substantial false-positive rates at early time points, while latent modeling suggested more stable cognitive-state patterns and different reversion dynamics (22). False positives can trigger stigma, anxiety, and cascades of referrals and testing; false negatives delay risk-factor management, planning, and access to interventions. Counterarguments deserve attention: fixed cut-offs are simple, and simplicity can be equitable when resources are scarce. Yet a simple rule that is systematically wrong for specific groups is not “resource sensitive” so much as “error efficient.” Equity requires that simplicity be achieved through better calibration and better pathways, not through pretending that one threshold fits all.

## From single cut-offs to calibrated, fair, and usable decisions

Equitable MCI care should treat screening scores as measurements with uncertainty, not verdicts. Operationally, we structure threshold governance around three pillars: calibration, transparency, and fit-for-purpose design (23–28). Instrument choice already demonstrates why, and the problem is not specific to MoCA alone. Parallel concerns about education effects, cultural loading, ceiling effects, and calibration are also reported for MMSE, Clock Drawing, and digital screening tools, suggesting that the core problem lies in how thresholds are used across instruments rather than in one test alone (8, 12, 23, 27). In a large Chinese population study comparing MMSE and MoCA, MoCA captured cognitive heterogeneity with less ceiling effect and yielded higher apparent MCI prevalence than MMSE (23). This can improve sensitivity, but it also means that a uniform cut-off can inflate “impairment” in groups whose prior educational opportunities reduce test familiarity. Longitudinal comparisons using Alzheimer's Disease Neuroimaging Initiative (ADNI) data show that MMSE and MoCA differ in how they track cognitive change over time, reinforcing that a single cross-sectional threshold is a weak proxy for risk trajectory (24). A fairer approach treats screening as the first step in a staged decision, where the second step may be repeat testing, domain-specific assessment, functional evaluation, or biomarker consideration depending on risk and patient goals.

Important elements of threshold governance have already been implemented, even if the term itself has not been used. Clinical recalibration of MoCA cut-offs to reduce spectrum bias in old age psychiatry, the development of demographically adjusted normative data in large multisite studies, and psychometric proposals for gray-zone interpretation all show that services already modify threshold use when fixed rules perform poorly in practice (1, 4, 29). These examples should be understood as partial implementations rather than a fully articulated governance model. The framework proposed here brings those strands together by linking local calibration, uncertainty-aware interpretation, and routine audit of downstream consequences.

Pillar 1 — Calibration: align norms and decision thresholds with the population and setting in which the test is used. Health systems should prefer demographically appropriate norms where available and should invest in building local norms when they are not. Calibration also includes redesigning measures to reduce educational and cultural loading. Short forms of the MoCA built using item response theory and adaptive-testing analytics illustrate how psychometrics can increase efficiency while preserving discrimination, but such designs still require fairness evaluation across language and education strata (25). Technology can widen access, but it can also widen bias. A Cochrane review of self-administered cognitive assessment tools concluded that evidence is insufficient to recommend any single tool and emphasized the need to understand how thresholds vary by setting, language, and education (26). A systematic review of touchscreen cognitive tools similarly noted promise for early detection but highlighted that evidence is concentrated in English-speaking, high-income settings, leaving major gaps for diverse populations (27). Remote self-administered cognitive screening in a national probability sample shows the feasibility of broader normative infrastructures, yet it also raises the responsibility to evaluate digital equity and measurement invariance across subgroups (28).

Pillar 2 — Transparency: make uncertainty explicit through gray zones and action thresholds linked to decision stakes. Clinicians should replace single cut-offs with explicit gray zones and action thresholds linked to decision risk. A psychometric analysis of the MoCA proposed an executive-function score and a “grey-zone” approach intended to represent uncertainty and reduce overconfident dichotomies (29). Table 1 provides a complementary workflow that aligns the stringency of decision thresholds with the stakes of the decision, and it specifies equity safeguards such as literacy screening, interpreter use, sensory checks, and subgroup auditing of false-positive and false-negative rates. This approach supports constructive counterarguments: it preserves pragmatic triage, but it acknowledges uncertainty and reduces predictable disparate error.

**Table 1:** Applying threshold governance in brief cognitive screening for MCI: common failure modes, immediate safeguards, and minimum audit measures for clinical use.

Decision stage	Common equity risk	Immediate safeguard	Minimum audit measures
Initial screening in primary care	Education/literacy-driven false positives; high reserve false negatives	Document language, education quantity and quality, literacy proxy; ensure sensory accommodations; treat score as triage not diagnosis	Subgroup referral rates; proportion receiving second-step assessment; false-positive proxies (rapid “normal” reclassification)
Interpreter-mediated or non-native language testing	Language discordance misread as impairment	Use validated local-language version; trained interpreter; avoid *ad-hoc* translation	Language-by-version performance; missingness and “unable to complete” rates
Use of culturally loaded items (abstraction, verbal fluency, clock drawing)	Cultural familiarity drives score more than neuropathology	Prefer culturally adapted versions; corroborate with functional history and informant report	Item-level Differential Item Functioning (DIF) signals where available; subgroup item performance patterns
Longitudinal monitoring with repeated screens	Practice effects and measurement non-invariance produce apparent change	Use consistent versioning; interpret change with context (health events, depression, sleep); confirm with domain testing when change is small	Subgroup trajectories; retest intervals; distribution of “improvement” vs. “decline”
Trial or therapy eligibility tied to score	Gatekeeping amplifies upstream disparities	Use staged confirmation (functional, domain testing, biomarkers when appropriate); avoid rigid single-score exclusion	Eligibility-by-subgroup; reasons for exclusion; time-to-confirmation
Remote/digital screening	Digital divide and device familiarity distort thresholds	Provide supported administration option; validate thresholds in target populations	Completion rates, device type effects, subgroup calibration error

The workflow is intended for sequential use in primary care or referral pathways; at each stage, a low or borderline score should trigger a context check and, where needed, a second-step assessment rather than an immediate diagnostic label.

Pillar 3 — Fit-for-purpose design: use instruments and norms that are built for diverse literacy, language, and cultural contexts rather than retrofitted cut-offs. A recent systematic review on identifying MCI and dementia in illiterate and low-educated populations concludes that many standard tools and thresholds fail precisely where the dementia burden is rising, and it calls for instruments and norms designed for those contexts rather than retrofitted cut-offs (30). Equitable MCI care therefore requires both technical humility and policy ambition: measure cognition in ways that respect lived context, and then use the results to expand, not restrict, access to timely evaluation and support.

Taken together, these three pillars are best understood as a quality-improvement loop rather than a one-off technical fix: calibrate the measure, make uncertainty explicit at the point of decision, and then audit downstream consequences to recalibrate. The aim is not to locate a single “correct” cut-off, but to build a thresholding pathway whose errors are visible, correctable, and proportionate to the clinical stakes. In the Discussion, we outline how this can be implemented in routine pathways without turning screening into an open-ended testing cascade.

## Discussion

Fixed cognitive cut-offs have become invisible policies in MCI care: they decide who is “in” and who is “out.” Because education, culture, comorbidity, and access are socially patterned, cut-off-based classification predictably reproduces those patterns. The way forward is not to abandon brief testing. It is to govern thresholds deliberately through calibration, staged decisions proportional to risk, and transparent auditing of disparate error. The ultimate equity test is simple: does the thresholding pathway reduce the chance that schooling, language, or access determines who gets timely MCI care?

In primary care, the next step will often be modest and standardized. Clinicians should first confirm sensory inputs (hearing and vision), check language concordance and the need for an interpreter, and address modifiable barriers such as pain, sleep disturbance, mood, medication effects, intoxication, or delirium risk. They can then either repeat testing at an agreed interval or move to a more targeted second-step assessment, such as domain testing, informant history, or a functional screen, before referral. Ownership should be explicit. The first-stage screen can sit with the General Practitioner (GP) team, while the second step can be protocolized and shared through practice nurse- or pharmacist-supported review of reversible contributors, structured informant tools, and timed follow-up testing, with clear triggers for memory service referral. Without this operational clarity, a call for calibration risks being interpreted as “do more testing” rather than “do the right testing, for the right reasons, in the right sequence.”

In feasibility terms, threshold governance does not require every borderline result to trigger specialist neuropsychology or biomarker testing. A proportionate primary care model is more realistic: most borderline results can be managed through repeat testing, reversible-cause review, medication review, and informant or functional assessment, while only persistent, progressive, or high-risk cases proceed to memory services or more advanced investigation. The main resource requirements are modest but real: staff training, access to validated language versions, interpreter support, structured follow-up capacity, and a small audit dataset. These should be treated as quality-assurance requirements for fair screening rather than as a mandate for universal escalation.

Equity also depends on how uncertainty is communicated. The harms of misclassification are asymmetric and socially patterned: false positives can create stigma, anxiety, and cascades of investigation; false negatives can delay vascular risk optimization, safety planning, and support. A gray zone should therefore be framed as a clinically meaningful state—“not normal, not diagnostic”—with a planned action rather than a vague doubt. Simple, uncertainty-aware language can protect trust while preserving momentum: for example, “This score is a starting point, not a diagnosis,” “Today's result is in the borderline range, so we will check and address anything that can affect performance and repeat the assessment,” and “If there is genuine change over time or functional impact, we will escalate the assessment.” Communication is not an optional extra; it is part of making staged decisions workable and acceptable.

Two cautions are important. First, demographic adjustment can be misused to normalize unequal education and to justify lower expectations for marginalized groups. Equity-minded thresholding must therefore be paired with explicit commitments to upstream improvement, including adult literacy support, interpreter services, sensory screening, and access to confirmatory evaluation. Second, clinicians worry that probabilistic language will paralyze decision-making. In practice, gray zones can do the opposite by clarifying what to do next: repeat testing, broaden assessment, or monitor change over time. A minimum viable equity package is feasible today: record language and education quality, correct modifiable barriers before testing, avoid labeling from one score, and track outcomes to recalibrate thresholds. These steps align accuracy with justice and help protect trust in MCI diagnoses.

Services can monitor a small set of core measures: completion rates, including “unable to complete” and why; referral and acceptance rates; yield of confirmatory assessment; time to second-step assessment and time to diagnosis; and short-term reclassification patterns. Rapid return to “normal” after barrier correction can be used as a pragmatic proxy for avoidable false positives. Crucially, these should be reviewed across relevant strata (language concordance, education/literacy proxies, deprivation, sensory impairment, and multimorbidity), alongside patient-reported understanding and distress after testing. The aim is not to perfect a number, but to make thresholding accountable: if one group repeatedly pays the price of a threshold—through excess false positives, excess false negatives, or slower access to confirmatory pathways—then the pathway, not the patient, is what needs to change. A simple service-level audit cycle could review these measures quarterly and trigger recalibration when one subgroup shows persistent excess false-positive proxies, excess false-negative proxies, low completion, or delayed movement to second-step assessment.

One practical way to operationalize threshold governance is to treat cut-off use like other safety-critical decision rules: assign named ownership, agree a small dashboard of metrics, and set a review cycle in which thresholds and pathways can be adjusted when disparities persist. This should be a shared responsibility across primary care, memory services, and neuropsychology, ideally with patient and public input so that recalibration reflects both measurement performance and lived experience. Even modest governance—such as quarterly review of completion, reclassification, and time-to-care across key strata—can prevent thresholds from hardening into unexamined gatekeeping.

Ultimately, cut-offs do not merely classify cognition; they shape access to further assessment, support, and opportunity. Recognizing thresholds as governance reframes fairness as a service responsibility: programmes should be able to justify the cut-offs they use, demonstrate how they perform across populations, and ensure that borderline results trigger a planned pathway rather than exclusion. In this sense, threshold governance is not an optional refinement—it is a practical way of aligning cognitive screening with both clinical accuracy and justice.
